# Strategies for reducing out of pocket payments in the health system: a scoping review

**DOI:** 10.1186/s12962-021-00301-8

**Published:** 2021-08-04

**Authors:** Faride Sadat Jalali, Parisa Bikineh, Sajad Delavari

**Affiliations:** 1 Student Research Committee, Shiraz University of Medical Sciences, Shiraz, Iran; 2 Health Human Resources Research Center, School of Health Management and Information Sciences, Shiraz University of Medical Sciences, Shiraz, Iran

**Keywords:** OOP, Out of pocket, Health policy, Health system, Financing, Scoping review

## Abstract

**Background:**

Direct out-of-pocket payments (OOP) are among the most important financing mechanisms in many health systems, especially in developing countries, adversely affecting equality and leading vulnerable groups to poverty. Therefore, this scoping review study was conducted to identify the strategies involving OOP reduction in health systems.

**Methods:**

Articles published in English on strategies related to out-of-pocket payments were Searched and retrieved in the Web of Science, Scopus, PubMed, and Embase databases between January 2000 and November 2020, following PRISMA guidelines. As a result, 3710 papers were retrieved initially, and 40 were selected for full-text assessment.

**Results:**

Out of 40 papers included, 22 (55%) and 18 (45%) of the study were conducted in developing and developed countries, respectively. The strategies were divided into four categories based on health system functions: health system stewardship, creating resources, health financing mechanisms, and delivering health services.As well, developing and developed countries applied different types of strategies to reduce OOP.

**Conclusion:**

The present review identified some strategies that affect the OOP payments According to the health system functions framework. Considering the importance of stewardship, creating resources, the health financing mechanisms, and delivering health services in reducing OOP, this study could help policymakers make better decisions for reducing OOP expenditures.

## Introduction

Nowadays, spending on health is rising, accounting for 10% of global gross domestic product (GDP). Government expenditures, out-of-pocket payments (OOPs), and sources like voluntary health insurance, employer-provided health programs, and activities by non-governmental organizations are all included in health spending [1].

As defined by the World Health Organization (WHO)*,* OOP expenses are the individuals’ direct payments to healthcare providers at the time of service use [2]. OOPs, include purely private transactions (payments made by individuals to private doctors and pharmacies), official patient cost-sharing (user fees/copayments) within defined public or private benefit packages, and informal payments (payments beyond the prescriptions entitled as benefits, both in cash and in-kind). Therefore, OOPs may be explicitly some part of a policy or can occur through market transactions, or both [3].

OOP health expenditures may increase whenever households opt to access and receive health services but are not protected against high payments since medical costs are high. They do not have access to insurance coverage and other safeguards against OOPs [4]. The following factors significantly affect OOP health care costs: increased patient cost-sharing, development of high-deductible health care plans, and more use of costly biologic or designer drugs. OOP payments are not an efficient way of financing health care and may negatively affect equity and cause vulnerable groups to experience poverty [5]. High OOP medical costs can use up financial savings and damage credits and have a negative impact on the quality of life, medication adherence, and different health outcomes [6].

A new report by the World Bank Group stated that OOP payments accounted for a non-negligible part of total health care expenditures in Central and Eastern European countries. Also, Patients in developing countries spent half a trillion dollars each year (over $80 per person) out of their own pockets to receive health services [7]. Unfortunately, such expenses significantly harmed the poor [8]. The more the health sector grew, the less reliant it would be on OOP spending. The total OOP spending increased at least twice as much in low- and middle-income countries during 2000–2017 and reached 46% in high-income ones. However, its growth was slower than that of public spending in all income groups [9]. According to Adam Wagstaf (2020), OOP expenditures changed significantly within income groups, ranging from $32 in Sweden to $1200 in Switzerland in the high-income groups, and from six dollars in Madagascar to $100 in Cambodia, Haiti, and Nepal in the low-income ones [10].

There have been health financing policy reforms and measures in several countries recently to deal with the concerns over high OOP payments. While there is no remedy, available information suggests that having well-designed policies and strategies can help countries reduce OOP and its adverse effects successfully [2, 11]. In general, reforms can apply some key strategies to abolish user fees or charges in public health facilities and exempt specific community groups such as the poor and the vulnerable, and pregnant women and children from official payments. They should also exempt some health services such as maternal and child care from official payments and deliver them free of charge [12].

Due to the lack of resources, implementing effective policies can protect households against the common and high costs of the health system. To date, no known study has reviewed the proposed appreciate strategies for reducing OOP health payments worldwide. So, the present study aims to investigate strategies of reducing OOP payments in the health system through scoping review studies between 2000 and 2020. This review can help decision-makers learn from the effective experiences of other countries in reducing OOP health payments.

### Materials and methods

This study was carried out based on the Joanna Briggs Institute scoping review method as a framework [13], and a comprehensive systematic scoping review was performed to explain the strategies that could effectively reduce OOP health expenditures around the world. A defined question based on the PCC (Population, Concept, and Context) elements was raised at the first stage. All the countries in the world (Population), strategies and policies that affected OOP health expenditures (Concept), and all health systems having OOP payments (Context) were included in the question.

The second stage dealt with the target population, which comprised all the studies related to “Out-of-Pocket Expenditures” in various countries. To this end, all related studies conducted since 2000 were retrieved through the research strategy (Table 1).

**Table 1:** The search strategy of the research

Search strategy	
Databases: PubMed, Scopus, ISI Web of Science, Embase (2000–2020)	
Limits: Language (resources in English) and date (published after 2000)	
Date: up to November 25, 2020	
Strategy: #1 AND #2 in title and abstract	
#1	“Out-of-Pocket Expenditure” OR “Out-of-Pocket Payment” OR “Out-of-Pocket Cost” OR “Out-of-Pocket Spending” OR “Out-of-pocket health spending” OR “Out of Pocket Expenditures” OR “Out-of-Pocket Expenses” OR “OOP” OR “Out of pocket”
#2	Strategy OR intervention OR policy

Thus, the original English keywords appropriate to the research objective were first selected based on the comments of the research team and the keywords used in available related studies. Then, PubMed, Scopus, ISI Web of Science, and Embase databases were searched. It was decided to identify all the articles with at least an English abstract indexed in one database.

**Fig. 1:**
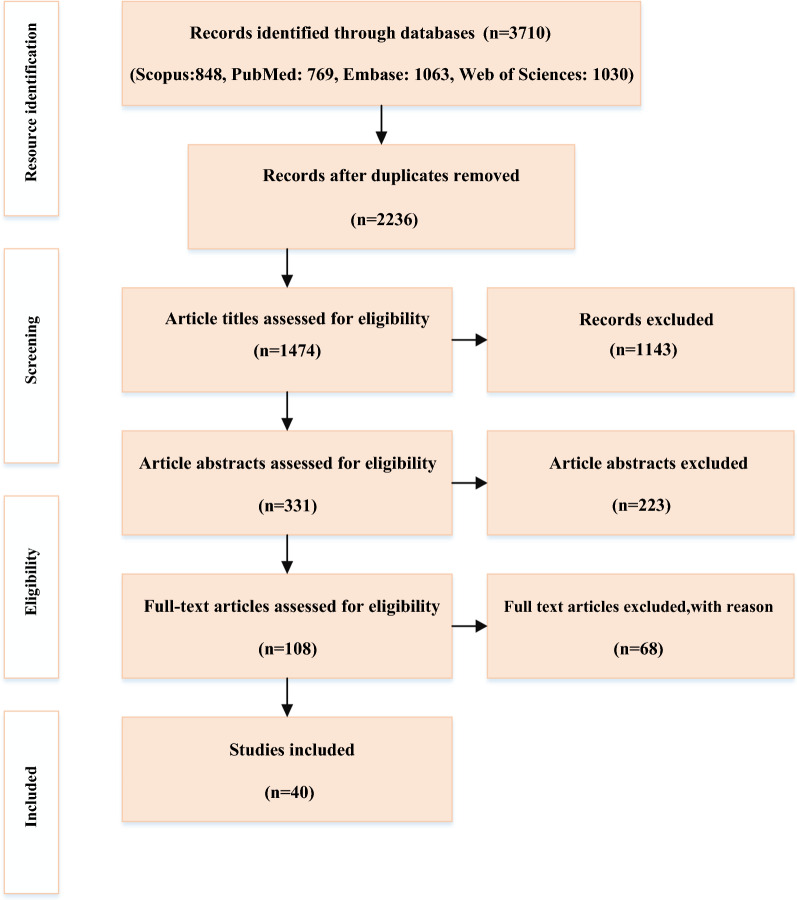
Preferred Reporting Items for Systematic Reviews and Meta-Analyses (PRISMA) flow diagram for thescoping review process

The selection of the relevant studies was carried out in the third stage. First, 3710 articles were indexed in all databases. After deleting duplicates, 1474 English-language articles were selected for review. Then, 223 articles were excluded from the list after reviewing the titles and abstracts, and 108 were chosen to review the full-text, and finally, the research team chooses 40 papers (Fig. 1). It is worth mentioning that all of the research processes and selection of the papers were conducted by two researchers independently (FSJ and PB), and a third researcher was responsible for reaching consensus if necessary (SD). Also, the protocols and review studies were not included in the present research. Finally, the Critical Appraisal Skills Program (CASP) tool was used to evaluate the quality of the original articles since it worked as a guide to cover the essential areas for critical appraisal of articles effectively.

In the last stage, the data were extracted from each study using the data-charting form (Appendix Table 4) and were collated and classified according to the thematic analysis provided.

In stage four, the data-charting form was used to extract data from each study (Appendix Table 4). Then, the collected data were collated and classified according to the thematic analysis in the last stage.

## Results

The findings resulted from the analysis of 40 studies were summarized in Appendix Table 5. Among these studies, 20 (50%), 13 (32.5%), four (10%), and three (7.5%) studies belonged to Asian, American, European, and African countries, respectively. Furthermore, 22 (55%) and 18 (45%) belonged to developing and developed countries.

Other findings show that four main factors have been emphasized as the effective factors on reducing OOP payments in the health systems, including Health system stewardship, creating resources, the health financing mechanisms, and delivering health services (Table 2).

**Table 2:** Component affecting on out of pocket reducing

Main components	Subcomponents	References	Reference evidence
Stewardship	Regulatory	[5, 14–31]	-Developing clinical guidelines [17] -Pro-poor health financing policy focusing on financial protection not only for those close to the poverty line, but also those who are already below it in both rural and urban areas [5] -Inclusion of private providers in the system [29]
	Implement regulations	[15, 16, 18–20, 22, 23, 30–42]	-Broadly implement DRGs and refine payment systems [33] -Universal health insurance coverage programs [28] -Create patient transport system in remote locations [38]
	Regulatory monitoring	[15, 18, 27, 29, 36–38, 43]	-A need for federal and state policymakers to reexamine how state agencies are applying the cost-sharing protections for contraception under Medicaid and Medicaid managed care plans [27] -Ensuring more investment for health from social health insurance and/or tax-based government funding [36]
Creating resources	Facilities	[5, 15, 32, 39, 44]	-The need for availability of drugs and medical supplies at the public facility [15] -Improving access to healthcare facilities like diagnostic test [32] -Telehealth (on-line video consultation) [44]
	Personnel	[17, 20, 27, 45, 46]	-Training the physicians [17] -Physicians should develop the habit of using brief just-in-time interventions at the point of prescription ordering [45]
Financing	Revenue collection	[5, 19, 29, 42]	-Innovative financing mechanisms on the collection side to reduce the intensity of poverty [5] -Exemption process of fees for the poor, disabled, and disadvantaged [19]
	Pooling	[5, 20, 21, 24, 30, 31, 37, 42, 43, 46–48]	-Mobilizing OOP payments on a pre-paid basis through formal or community-based risk-pooling schemes [24] -Enrolment into health insurance [37] -Basic Insurance Scheme (BIS) (Retired workers are exempt from premium contributions, and the cost of their contributions is to be borne by their former employers) [48]
	Purchasing	[5, 14, 18, 33, 49]	-Diagnosis-related group (DRG)–based payment system [49] -Performance-based payment [14]
Delivering services	Prevention	[5, 16, 24, 27, 28, 31, 50, 51]	-Screening and in situ treatment of precancerous cervical lesions for women between 25 and 55 years old [16] -The National Programme for Prevention and Control of Cancer, Diabetes, Cardiovascular Disease and Stroke [31] -Breast cancer screening [51] -Integrating the prevention and control of oral diseases into universal health insurance coverage programs [28]
	Treatment	[6, 15, 20, 27, 32, 39, 45, 50]	-The improved effectiveness of services [15] -Limiting X-ray and ultrasound orders [50] -Switching to a generic form of intervention [6]

As it derives from Table 2, 31 (77.5%) articles pointed to the “role of the healthcare system stewardship” as one of the main components in reducing OOP payments. The three subcomponents under the tutelage include legislation (40.5%), legislation implementation (42.5%), and effective monitoring (17%).

The second most referred main component belonged to the "health financing mechanisms " with 18 articles (45%) and three subcomponents, namely revenue collection (19%), pooling and Resource management (57%), payment and purchasing (24%).

The number of article on “delivering health services” were 15 (37.5%). It has two sub-components of preventive services (50%), and treatment (50%) have been considered as one of the main components affecting the reduction of OOP payments in health systems.

“Creating Resource” with ten articles (25%) and two sub-components, namely the physical resources (50%), human capital investment, and training (50%), also have the least referred in the articles.

As Table 3 shows, developed and developing countries have implemented various strategies to reduce OOP. Developed Asian countries have applied medical subsidy, universal health coverage, Choosing the right pharmacy, requesting inexpensive generic drugs by patients, the inclusion of dental care coverage in health insurance packages, control strategies drug price, performance-based payment, eliminating OOP costs for methods of contraception, choose a brand-name drug with a generic equivalent, free screening, drug coupons, promoting the quality of primary care services, ordering by physicians and telehealth as effective strategies in reducing OOP payments. Government support of public health insurance program, subsidy program for diseases with high economic burden, prevent and control chronic diseases, training the physicians, developing clinical guidelines, universal health coverage, diagnosis-related group (DRG) based payment system, expanding the dental health reform, providing care closer to home, Insurance for children, students, the elderly, the disabled, and other unemployed populations in urban regions groups not covered by basic health insurance catastrophic disease insurance, increase the efficiency and quality of care, free treatment to the vulnerable segment of the population, clear the system from informal payment, -innovative financing mechanisms on the collection, pooling and purchasing side, free gynecologic screening and discharging patients earlier are some strategies that Asian developing countries such as Iran, India, China, Bangladesh and the Philippines and African countries such as Ethiopia and Ghana have implemented to reduce their OOP payments.

**Table 3:** Implemented interventions in developed and developing countries

Interventions in developed countries	Asia	-Medical subsidy for children -Universal health coverage; this is achieved through public health insurance
	America	-Prescription content and Choosing right pharmacy -Writing 90-day prescriptions and choosing the lowest-cost generic drugs by Prescribers -Requesting inexpensive generic drugs by patients -Control strategies drug price -Employ centralized price and comparative and cost-effectiveness research for determining price ceilings -Universal health coverage -Strategies Involving Care-Plan Changes: Changing to lower-cost alternative intervention, Switching to generic form of intervention, changing dosage/frequency of intervention -Strategies not involving care-plan changes: Changing logistics of care, ––Facilitating co-pay assistance or coupons, Providing free samples, changing or adding insurance plans -Innovation in drug pricing to include value, the introduction of performance-based payment -Removal of consumer cost-sharing for contraceptives -Federal coverage in eliminating OOP costs among privately insured women for at least some methods of contraception -Improve private health plans -Choose a brand-name drug with a generic equivalent -Free breast cancer screening -Drug coupons for multiple sclerosis -Adults individual insurance -The Medicare insurance -Prioritize public financing of services for the poor -Promoting the quality of primary care services; -Mobilizing OOP payments on a pre-paid basis through formal or community-based risk-pooling schemes -Using brief just-in-time interventions at the point of prescription ordering by physicians -Discontinuing nonessential medicines -Use of an over-the-counter medicine as a substitute -Refer the patient to a public aid agency or social worker
	Oceania	-Telehealth (on-line video consultation)
	Europe	-Inclusion of dental care coverage in health insurance packages -Integrating the prevention and control of oral diseases into universal health insurance coverage programs
Interventions in developing countries	Asia	-Government support of public health insurance program -Subsidy program for diseases with a high economic burden -Prevent and control chronic diseases -Training the physicians -Developing clinical guidelines -Universal health coverage -Diagnosis-related group (DRG)–based payment system -Expanding the dental health reform -Providing care closer to home -Improve the effectiveness of services -Regularly updating the essential list of drugs according to need of patients -Mandatory social insurance program for urban employees -Insurance for children, students, the elderly, the disabled, and other unemployed populations in urban regions groups not covered by basic health insurance -Catastrophic Disease Insurance -Increase the efficiency and quality of care -National Program for Prevention and Control of Cancer, Diabetes, Cardiovascular Disease, and Stroke -Free treatment to the vulnerable segment of the population for the treatment of cancer and heart diseases -Create patient transport system in remote locations -Fees exemption for the poor, disabled, and disadvantaged -Public and private insurance -More investment for health from social health insurance and tax-based government funding -Inclusion of private providers in the system -Decrease and even eliminate the copayments for those at low-income levels -Clear the system from informal payments -Innovative financing mechanisms on the collection, pooling, and purchasing side
	Africa	-Free maternal health care policy -Screening and in situ treatment of precancerous cervical lesions for women between 25 and 55 years old and clinical screening for breast cancer at age 15 -Limiting prescription of brand-name drugs, x-ray and ultrasound orders, screening tests, advanced lab tests, ward/ICU admission, surgery -Discharging patients earlier -Refuse expensive drug requested by patients or families -Reducing informal fees

## Discussion

Overall, the results showed that four main components—health system stewardship, financing mechanisms, service delivery, and creating resources—have been effective in reducing OOP payments in health systems of different countries. This category is similar to the functions introduced by the WHO report in 2000 [52].

Legislation, legislation implementation, and effective monitoring are considered as proposed subcomponents of the stewardship. The results of many studies have shown that health care governance around the world can reduce household health expenditures by legislation. For example, Rahman et al. (2020), in their research in Bangladesh, stated that health care governance, strengthening the rules and regulations related to care subsidies by public health centers, counseling and planning clinics for parents, and Community-based health centers for low-income consumers and patients with high economic burden can play an important role in reducing OOP healthcare costs [31]. Sarnak et al. (2017) cited federal government negotiations and legislation on the announcement of centralized prices. They approved drug ceiling rates in the United States as one factor in reducing OOP payments [22].

Ensuring implementation and monitoring the correctness of the laws by health system governance can also help reduce OOP payments. Several studies have identified the implementation of laws and programs related to global health care coverage as a way to protect households from these expenditures [22, 23, 28, 35, 36].

Control on the efficiency and quality of care and payment systems [18], Careful monitoring to clear the informal payments [29, 37] and ensuring the supply and availability of essential medicines [38] is also helpful in this regard.

According to the present study results, by investing in human capital investment and training and physical facilities, OOP payments can also be reduced. Providing the infrastructure for online video consultation in Australia [44], improving access to health facilities in India [32], physicians' training on various fields in Iran [17] and the United States [45], had been reported as effective strategies in reducing OOP.

On the other hand, the lack of financial protection has been recognized as a health system disease. OOP payments are one of the major financing mechanisms in many developing countries and put the poor's greatest pressure. Adequate financing and its functions, including revenue collection, risk pooling and purchasing, are introduced as the most important mechanisms in reducing the share of direct OOP payment [53]. For example, Aryeetey et al. (2016), in their study in Ghana, stated that enrolment into health insurance would reduce OOP payments by 80% [37].

Several studies have also expanded the intensity and health insurance coverage for dental services [21]، rare and incurable diseases treatment [31], and mentioned the support for vulnerable groups as effective factors in this regard [42]. A study in India found that using new methods of health financing to collect, pooling and purchasing would reduce the severity of poverty and OOP payments [5], including pay for performances [14] and diagnosis-related groups (DRGs) payments [18, 33, 49].

Also in this research, the provision of prevention and treatment services have been included as two sub-components of providing health services. Some studies have shown that taking precautionary measures can prevent many OOP payments in the future. Meda et al. (2019) stated that the implementation of screening programs for gynecological diseases in reproductive age would prevent cancer in later years and thus will lead to individual financial protection [16].

The results of a study by Kastor et al. Showed that launching national prevent and control cancer, diabetes, cardiovascular disease, and stroke programs in India significantly reduces OOP payment [31].

It is worth mentioning that the studies obtained from the present study showed that in addition to preventive services, the providers' behaviors and actions are also effective in reducing OOP payments. physicians can replace generic drugs with brand drugs in their prescriptions [20, 39, 45] Limiting diagnostic-therapeutic tests and surgeries and preventing unnecessary admissions in special intensive care wards and alternative interventions, discharge patients quickly [50] And improve the quality and effectiveness of services [15, 18], play an effective role in reducing OOP payments.

Also, as this study shows, employing cost-effectiveness research for determining price ceilings, dental care coverage in health insurance packages, control strategies drug price, and on-line video consultation are some strategies implemented in developed countries. But developing countries have implemented strategies, such as government support of public health insurance programs, subsidy programs for diseases with high economic burdens, training the physicians, eliminate informal payments, and discharging patients earlier. Strategies such as free screening programs, universal health coverage, pay for performance, promoting the quality of care services and replacing the brand drug with generic have been common in both developed and developing countries. The reason for these differences can be sought in factors such as the medical capacity of countries, per capita government funding, different patterns of disease, the governing system, and the health financing system. A study in Iran cited economic factors, policy factors, social support organizations, insurance, cost of health services, tariffs, health services organizations, providers and consumers’ behaviors, and epidemiological conditions as factors influencing OOP health payments [54].

It should be noted that this study by a research team has reviewed articles related to effective solutions to reduce OOP payments in the health systems of different countries. The search strategy consisted of four electronic databases, and two independent researchers evaluated each article.

This study faces several limitations including limitations related to databases and search strategies by researchers. As well the suggested strategies were not surveyed regarding to effectiveness or cost. Therefore, more studies are needed to check the cost and effectiveness of suggested strategies for reducing OOP.

### Conclusion

One of the most important characteristics of successful countries in providing maximum health for their communities is the rationality of the financing method and maximizing the share of the public sector in the share of OOP payments in health services so that people feel comfortable when the disease occurs. In case of disability and poverty, do not give up health services.

The present review identified the importance of each health system's functions that affect the reduction of OOP payments. Given that OOP payments are the worst form of financing in any health system, the strategies proposed and successfully implemented worldwide must be considered by policymakers when making future decisions to target health systems. Approach their goals, which include promoting health, increasing accountability, and equitable financial participation.

## Data Availability

Data of this research is available and could be sent upon contact with the corresponding author.

## Appendix 1

See Tables 4 and 5.

**Table 4:** A draft chart of data extraction

General information	
Title of the manuscript	
Article No	Language
Year of the publication	First author
Place (Country)	Corresponding
Type of article	Journal name
Article characteristics	
Aims of the study	
Study approach study design	
Methodology	
Sampling method study environment	
Data collection study population	
Data analysis sample size	
Results	
Main results	
Conclusion	
Recommendations	
Limitations

**Table 5:** Selected Studies on out of pocket reducing strategies

N	Title	Author	Year	Place	Type	Participants	Results
1	Disease-specific out-of-pocket healthcare expenditure in urban Bangladesh: a Bayesian analysis	Md. Mahfuzur Rahman	2020	Bangladesh	Research Article	Urban areas of Rajshahi 1593 households	-Reducing out of pocket payments, after implementing government supported/public not private health insurance program in LMIC countries, particularly Vietnam, China, and the Philippines -To avoid unpredictable medical expenses, the Government of Bangladesh should start health insurance program in its health-financing unit -Implementation of a subsidy program for diseases with high economic burden like renal diseases, cancer, and heart diseases -More attention to prevent and control chronic diseases
2	Over Medication and Waste of Resources in Physicians’ Prescriptions: a Cross Sectional Study in Southwestern Iran	Mohammadreza Heydari	2020	Iran	Research Article	392 physicians	-Training the physicians -Developing clinical guidelines
3	The effect of a community-based health insurance on the out-of-pocket payments for utilizing medically trained providers in Bangladesh	Jahangir A. M. Khan	2020	Bangladesh	Original Article	1292 (646 insured and 646 uninsured) households	-The CBHI scheme (community-based health insurance), and could push the country towards universal health coverage
4	Strategies to Reduce Out-of-Pocket Medication Costs for Canadians Living with Heart Failure	William F. McIntyre	2020	Canada	Original Article	Outpatient pharmacies in Hamilton, Ontario	-Prescription content, dispensing practice, and pharmacy choice can remarkably impact out-of-pocket costs for HF medications -Prescribers can reduce costs by writing 90-day prescriptions and choosing the lowest-cost generic drugs in each therapeutic class -Patients should request inexpensive generic drugs
5	Economic Implications of Chinese Diagnosis-Related Group–Based Payment Systems for Critically Ill Patients in ICUs	Zhaolin Meng, MMed	2020	China	Original Research	6679 critically ill patients who received intensive care in the 22 public hospitals)	-Chinese diagnosis-related group, (C-DRG)–based payment system achieved success in reducing OOP payments for critically ill patients by shifting the payment of OOP costs from FFS to DRG
6	Factors associated with disparities in outof-pocket expenditure on dental care: results from two cross-sectional national surveys	Orenstein, L	2020	Occupied Pelastine	Original Research Article	8465 households in 2014 and 8792 households	-Expanding the dental health reform and addressing barriers to preventive dental care -Expanding basic and supplementary dental health insurance
7	A new hope: from neglect of the health sector to aspirations for Universal Health Coverage in Myanmar	Alex Ergo1	2019	Myanmar	Original Paper	3648 households in Myanmar	-Confidence of services delivery are available at public facilities -The availability of services closer by -Improve effectiveness of services -Necessary drugs and medical supplies should be available at the public facility
8	Out-of-pocket payments in the context of a free maternal health care policy in Burkina Faso: a national cross-sectional survey	Ivlabèhiré Bertrand Meda	2019	Burkina Faso	Original Research	Women (n = 593) who had delivered or received obstetric care on the day of the survey	-Free maternal health care policy -Antenatal care, normal deliveries and EmONC, curative care during pregnancy and up to 42 days after delivery, treatment of obstetric fistulas, screening and in situ treatment of precancerous cervical lesions for women between 25 and 55 years old and clinical screening for breast cancer starting at age 15 -Improvements in the management and supply system of health facilities’ pharmacies
9	Financial risk protection at the bedside: how Ethiopian physicians try to minimize out of pocket health expenditures	Ingrid Miljeteig	2019	Ethiopia	Research Article	565 physicians	-Limiting prescription of brand named drugs/hospital drugs -Explaining alternatives and recommending affordable options -Limiting x-ray and ultrasound orders -Providing second best treatment -Limiting screening tests -Limiting advanced lab tests -Limiting ward/ICU admission -Discharging patients earlier than you wanted -Limiting surgery unless highly indicated -Delaying a treatment or test to see if possible to do without it -Referring patients to other less expensive options -Providing less frequent follow up of NCDs (chronic conditions) -Limiting CT or MRI orders -Not informing the patient about expensive options -Screening patient for dialysis -Refuse expensive drug requested by patients or families
10	Health financing strategies to reduce out-of-pocket burden in India: a comparative study of three states	Montu Bose	2018	India	Comparative Study Research Article	3917 households from TN, 2912 households in Rajasthan and 5019 households from WB	-Procuring medicine or regularly updating the essential list of drugs according to need of patients are urgently required in West Bengal -Improving access to healthcare facilities like diagnostic test etc -TN and Rajasthan health financing strategies
11	Reducing the medical economic burden of health insurance in China: Achievements and challenges	Dou, G. S	2018	China	Policy Forum	–	-Establish a mandatory social insurance program for urban employees -Establish the New Rural Cooperative Medical Scheme (NCMS) -Establish Urban Resident Basic Medical Insurance (URBMI) for Children, students, the elderly, the disabled, and other unemployed populations in urban regions groups not covered by basic health insurance -Increase government subsidies to NCMS and URBMI to an unprecedented scale -Launch Catastrophic Disease Insurance -Replace FFS with other forms of prospective payment (prospective payment systems such as the global budget payment system -DRG payments -Increase the efficiency and quality of care under current payment systems
12	Disease-specific out-of-pocket and catastrophic health expenditure on hospitalization in India: do Indian households face distress health financing?	Anshul Kastor	2018	India	Research Article	3,33,104 individuals from 65,932 households (36,480 rural and 29,452 urban households)	-The Ministry of Health and Family Welfare, Government of India launched the National Program for Prevention and Control of Cancer, Diabetes, Cardiovascular Disease and Stroke -Suggest for including treatment of cancer, heart diseases, and other rare and incurable diseases in the ambit of the health insurance coverage -Provide free treatment to the vulnerable segment of the population for the treatment of cancer and heart diseases -The coverage and the insurance amount of the RSBY need to be enlarged
13	Does public health system provide adequate financial risk protection to its clients? Out of pocket expenditure on inpatient care at secondary level public health institutions: causes and determinants in an eastern Indian state	Rout, S. K	2018	India	Research Article	239 inpatients	-Health system should ensure supply of essential medicines and create patient transport system in remote locations to reduce OOPE
14	The impact of out-of-pocket payments for dental care on household finances in low and middle income countries	Eduardo Bernabé	2017	London	Research Article	40 low and middle income countries (1,74,257 adults, aged 18 years and above)	-Inclusion of dental care coverage in health insurance packages -Integrating the prevention and control of oral diseases into universal health insurance coverage programs
15	Predictors of high out-of-pocket healthcare expenditure: an analysis using Bangladesh household income and expenditure survey, 2010	Azaher Ali Molla	2017	Bangladesh	Research Article	640 rural and 360 urban households	-Providing a safety net for low-income rural households and for elderly members -Control and prevention of chronic diseases -Universal coverage of healthcare -Launching some new types of safety net for the poor, the disabled and women -Exemption process of fees for the poor, disabled and disadvantaged
16	Paying for Prescription Drugs Around the World: Why Is the U.S. an Outlier?	Dana O. Sarnak	2017	U.S	Issue Brief	Pharmaceutical spending in high-income, industrialized countries	-Control strategies drug price -Employ centralized price, negotiations, national formularies, and comparative and cost-effectiveness research for determining price ceilings -Federal government should negotiate lower drug prices for Medicare beneficiaries -Universal health coverage
17	What Strategies Do Physicians and Patients Discuss to Reduce Out-of-Pocket Costs? Analysis of Cost-Saving Strategies in 1755 Outpatient Clinic Visits	Wynn G. Hunter	2016	America	Original Article	Patients with breast cancer, depression, rheumatoid arthritis visiting oncologists, psychiatrists, and rheumatologist	Strategies Involving Care-Plan Changes Changing to lower-cost alternative intervention Switching to generic form of intervention Changing dosage/frequency of intervention Stopping or withholding intervention Strategies not involving care-plan changes Changing logistics of care Facilitating co-pay assistance or coupons Providing free samples Changing or adding insurance plans
18	Can health insurance protect against outof-pocket and catastrophic expenditures and also support poverty reduction? Evidence from Ghana’s National Health Insurance Scheme	Genevieve Cecilia Aryeetey	2016	Ghana	Original	3000 households	-Enrolment into health insurance reduced household costs -Protective effect of the NHIS -Reduce informal fees
19	The financial burden of out-of-pocket expenses in the United States and Canada: How different is the United States?	Katherine E Baird	2016	Canada	Original Article	2,03,799 United States 60,313 Canada Households	-Expansion in insurance levels -The ACA’s to match the actuarial value of insurance to income, and to place more stringent limits
20	The Parity Paradigm: Can Legislation Help Reduce the Cost Burden of Oral Anticancer Medications?	Sheetal M. Kircher	2016	Chicago	Policy Perspective	–	-Innovation in drug pricing to include value, introduction of performance-based payment, and a shift from coverage based on the route of administration
21	Women Saw Large Decrease In Out-Of-Pocket Spending For Contraceptives After ACA Mandate Removed Cost Sharing	Becker, N. V	2015	United States	Original	Women ages 13–45 who were enrolled in private health insurance	ACA-mandated removal of consumer cost sharing for prescription contraceptives (pill and IUD) in non grandfathered insurance plans
22	Payment reform pilot in Beijing hospitals reduced expenditures and out-of-pocket payments per admission	Jian, W	2015	Beijing	Original	Shifting payment of 108 diagnoses or procedures from FFS payment to a DRG at six tertiary general hospitals	-Broadly implement DRGs and refine payment systems
23	Changes in out-of-pocket payments for contraception by privately insured women during implementation of the federal contraceptive coverage requiremen	Lawrence B. Finer	2014	America	Original research article	3207 women aged 18–39 years	-Federal coverage in eliminating out-of-pocket costs among privately insured women for at least some methods of contraception -Improve private health plans -Choose a brand-name drug with a generic equivalent -A need for federal and state policymakers to reexamine how the cost sharing protections for contraception under Medicaid are being applied by state agencies and Medicaid managed care plans -Subsidized care provided by publicly supported health centers, such as health departments, Planned Parenthood clinics and community health centers, to low-income clients
24	Understanding Patient Options, Utilization Patterns, and Burdens Associated with Breast Cancer Screening	Harvey, S. C	2014	America	–	–	-Breast cancer screening (patient recall for further diagnostic imaging or procedures) -Free breast cancer screening
25	Specialty Drug Coupons Lower Out-Of-Pocket Costs And May Improve Adherence At The Risk Of Increasing Premiums	Starner, C. I	2014	United States	Original	2,64,801 pharmacy’s prescriptions	-Drug coupons for multiple sclerosis or biologic anti-inflammatory drugs
26	Health-Related Financial Catastrophe, Inequality and Chronic Illness in Bangladesh	Md. Mizanur Rahman1	2013	Bangladesh	Original Article	Urban areas of Rajshahi 1593 households	-Implementing compulsory health insurance for salaried workers in both public and private sectors, and voluntary memberships for dependents, farmers and self-employed persons -Improving routine management of NCDs, to reduce the cost of chronic disease management, and incorporating chronic disease management into public services and health financing initiatives, to ensure that this expenditure is included in risk pooling and welfare initiatives and the high OOP payments associated with chronic illness -Incorporating ancillary services into basic care packages in public facilities
27	The impact of health insurance programs on out-of-pocket expenditures in Indonesia: an increase or a decrease?	Budi Aji	2013	Indonesia	Original Article	Indonesia Family Life Survey (IFLS) dataset covering 7224 households	This study showed that two large existing health insurance programs in Indonesia, Askeskin and Askes. The ability of programs to offer financial protection by reducing out-of-pocket expenditures is likely to be a direct function of their benefits package and co-payment policies
28	The health care system reform in China: eects on out-of-pocket expenses and saving	Atella, V	2013	China	Original Article	Individuals and households from 11 provinces and municipalities from Household Income Project surveys	-The third stage of the health care system reform, Basic Insurance Scheme (BIS). (The program is financed by premium contributions from employers and employees. Retired workers are exempt from premium contributions and the cost of their contributions is to be borne by their former employers. his program expands coverage to private enterprises and smaller public enterprises. Moreover, self-employed workers are allowed to enter the program.) -Public and private insurance prove to serve as a cushion against health risks
29	Practical aspects of telehealth: financial considerations	Loh, P. K	2013	Australia	Original	–	-Telehealth (online video consultation) save travel time for patients, careers and specialists, and can reduce out-of-pocket expenses
30	Out-of-pocket medical expenses for inpatient care among beneficiaries of the National Health Insurance Program in the Philippines	Tobe, M	2013	Philippines	Original	94,531 insurance claims	-Ensuring more investment for health from social health insurance and/or tax-based government funding, as well as shifting the provider payment mechanism from a fee-for-service to a case-based payment method, is essential -NHIP (National Health Insurance Program in the Philippines)
31	Community pharmacy-based medication therapy management services: financial impact for patients	Dodson, S. E	2012	America	Original Research	Medicare Part D members who had been previously identified as eligible for MTM services (128 patients)	-Patient participation in MTM services (Medication Therapy Management)
32	Impact of healthcare reforms on out-of-pocket health expenditures in Turkey for public insurees	Erus, B	2012	Turkey	Original Paper	Household Budget Survey	-Inclusion of private providers in the system -Decrease and even eliminate the co-payments for those at low-income levels -Clear the system from informal payments
33	Individual Insurance Benefits To Be Available Under Health Reform Would Have Cut Out-Of-Pocket Spending In 2001–08	Hill, S. C	2012	Rockville, Maryland	Original	Adults ages 26–64 with individual insurance, insurance through small employers, insurance through large employers	-Adults individual insurance reduce risk for high out-of-pocket spending and lower average out of-pocket costs for medical care and prescription drugs
34	Insured yet vulnerable: out-of-pocket payments and India's poor	Shahrawat, R	2012	India	Original	1,24,644 (45 346 urban and 79,298 rural) households	-No OOP payments for drugs, for inpatient and for outpatient visits—on impoverishment -Need to expand program benefits beyond inpatient care -Insurance schemes like the RSBY which focus on the poor are an important new initiative to reduce the impoverishing effects of OOP payment for health -Insurance schemes targeting the poor need to have a sufficiently wide coverage
35	Promoting Access and Reducing Expected Out-of-Pocket Prescription Drug Costs for Vulnerable Medicare Beneficiaries	Timothy W. Cutler	2011	California	Brief Report	Vulnerable Medicare beneficiaries	-Vulnerable Medicare beneficiaries should receive assistance from pharmacists and trained pharmacy students and enroll in the lowest-cost plans -The Medicare Part D benefits
36	Effect of a medical subsidy on health service utilization among schoolchildren: A community-based natural experiment in Japan	Miyawaki, A	2010	Japan	Original	Children who were in grades 1–6	-Medical subsidy for children (MSC) -Universal health coverage; this is achieved through public health insurance
37	Reducing out-of-pocket expenditures to reduce poverty: a disaggregated analysis at rural–urban and state level in India	Garg, C. C	2009	India	Comparative Study	1,20,000 household	-Rationalized drug policies (including free supplies) -Pro-poor health financing policy focusing on financial protection not only for those close to the poverty line, but also those who are already below it in both rural and urban areas; and innovative financing mechanisms on the collection, pooling and purchasing side to reduce the intensity of poverty -National Rural Health Mission (NRHM) provide quality health care to every household through its upgraded health infrastructure and provision of round the-clock health services
38	State Variations In The Out-OfPocket Spending Burden For Outpatient Mental Health Treatment	Zuvekas, S. H	2009	America	Original	Using data from the Medical Expenditure Panel Survey (MEPS)	-Consideration of policies related to the medications that account for two thirds of out-of-pocket spending -Manage costly medications through policies such as tiered and restrictive formularies, increased cost sharing, step therapy, and prior authorization -Encourage the use of effective generic medications, in place of expensive brand name medications
39	Health care-seeking behaviour and out-of-pocket payments in Tbilisi, Georgia	Gotsadze, G	2005	Georgia	Original	2500 households	Prioritize public financing of services for the poor, in particular through amending the Basic Benefit Package so that it better reflects the needs of the poor; -Promote the quality and utilization of primary care services; -Address the issue of rational drug use; -Consider mobilizing out-of-pocket payments on a pre-paid basis through formal or community based risk pooling schemes
40	Physician Strategies to Reduce Patients’ Out-of-pocket Prescription Costs	Alexander, G. C	2005	United States	Original Investigation	700 General internists and 700 cardiologists from the American Medical Association	-Physicians should develop the habit of using brief just-in-time interventions at the point of prescription ordering -Physicians should develop the habit of using brief just-in-time interventions at the point of prescription ordering -Switch from a brand-name to a generic drug -Give the patient office samples -Critically review medication list and discontinue nonessential medicines -Switch to a different brand-name drug within the same drug class -Prescribe a higher dose of medicine and tell the patient to split the tablets -Refer the patient to a pharmaceutical company assistance program -Recommend the use of an over-the-counter medicine as a substitute -Refer the patient to a public aid agency or social worker
